# Concepts of pathogenesis in psoriatic arthritis: genotype determines clinical phenotype

**DOI:** 10.1186/s13075-015-0640-3

**Published:** 2015-05-07

**Authors:** Oliver FitzGerald, Muhammad Haroon, Jon T Giles, Robert Winchester

**Affiliations:** Department of Rheumatology, St Vincent’s University Hospital, Elm Park, Dublin, 4 Ireland; Rheumatology Division, Department of Medicine, Columbia University College of Physicians and Surgeons, 630 West 168th Street, New York City, NY 10032 USA

## Abstract

This review focuses on the genetic features of psoriatic arthritis (PsA) and their relationship to phenotypic heterogeneity in the disease, and addresses three questions: what do the recent studies on human leukocyte antigen (HLA) tell us about the genetic relationship between cutaneous psoriasis (PsO) and PsA – that is, is PsO a unitary phenotype; is PsA a genetically heterogeneous or homogeneous entity; and do the genetic factors implicated in determining susceptibility to PsA predict clinical phenotype? We first discuss the results from comparing the HLA typing of two PsO cohorts: one cohort providing the dermatologic perspective, consisting of patients with PsO without evidence of arthritic disease; and the second cohort providing the rheumatologic perspective, consisting of patients with PsA. We show that these two cohorts differ considerably in their predominant HLA alleles, indicating the heterogeneity of the overall PsO phenotype. Moreover, the genotype of patients in the PsA cohort was shown to be heterogeneous with significant elevations in the frequency of haplotypes containing HLA-B*08, HLA-C*06:02, HLA-B*27, HLA-B*38 and HLA-B*39. Because different genetic susceptibility genes imply different disease mechanisms, and possibly different clinical courses and therapeutic responses, we then review the evidence for a phenotypic difference among patients with PsA who have inherited different HLA alleles. We provide evidence that different alleles and, more importantly, different haplotypes implicated in determining PsA susceptibility are associated with different phenotypic characteristics that appear to be subphenotypes. The implication of these findings for the overall pathophysiologic mechanisms involved in PsA is discussed with specific reference to their bearing on the discussion of whether PsA is conceptualised as an autoimmune process or one that is based on entheseal responses.

## Introduction and concepts of pathogenesis

Psoriatic arthritis (PsA) is a heterogeneous disease with diverse clinical and radiographic manifestations. However, the specificity reported in development of the Classification Criteria for Psoriatic Arthritis [1] strongly implies that there are indeed common features which facilitate the diagnosis of PsA. Nonetheless, patients often differ considerably from one another in the type of their clinical manifestations, including whether or not they exhibit dactylitis, enthesitis, asymmetric or symmetric sacroiliitis, or joint deformity, but also in the presence and type of joint damage on plain radiography. For example, in a recent cohort study of patients with long-established PsA, 56% showed no evidence of erosive disease after a mean follow-up of 19 ± 9 years, while the balance had erosive disease [2]. Diverse other varied radiographic features were present, including features such as new bone formation, osteolysis and ankylosis (Table 1). The basis for this heterogeneity had not been adequately explained. In this review, the two major hypotheses relating to disease pathogenesis are discussed in light of recent data from studies of human leukocyte antigen (HLA) genotypes in PsA [3,4] and a study on the relationship of genotype to phenotype [2].

**Table 1:** **Phenotypic heterogeneity in a cohort of 282 cases of psoriatic arthritis**

Age	55 ± 12
Male gender	134 (47)
Family history of psoriasis (*n* = 280)	175 (63)
Family history of PsA (*n* = 280)	45 (16)
Age of onset of psoriasis	28 ± 14
Psoriasis duration (years)	27 ± 14
Age of onset of PsA	35 ± 13
PsA duration (years)	19 ± 9
Years between psoriasis and PsA	6 (0 to 14)
Psoriasis before PsA	184 (65)
PsA before psoriasis	45 (16)
Psoriasis and PsA at same time	52 (18)
Nail disease	244 (79)
Pitting	96 (34)
Onycholysis	155 (55)
Peripheral arthritis	280 (99)
Polyarthritis	258 (91)
Oligoarthritis	21 (8)
Deformed joint count, if deformity	6 (4 to 12)
Enthesitis^a^	97 (34)
Dactylitis^a^	150 (53)
Sacroiliitis^a^	71 (25)
Asymmetric^a^	51 (18)
Symmetric^a^	18 (6)
Joint deformity^a^	183 (65)
Joint fusion^a^	83 (29)
Erosions^a^ (*n* = 281)	123 (44)
Osteolysis^a^ (*n* = 281)	41 (15)
Current PASI	1.2 (0.3 to 2.8)
Max PASI	3.8 (1.8 to 9.2)
Plaque psoriasis only	224 (79)
Psoriasis, nonplaque	59 (21)
Uveitis	9 (3)
Total on TNFi	176 (62)
Psoriasis requiring TNFi	32 (11)
PsA requiring TNFi	171 (60)
Number of TNFi, if on TNFi	1 (1 to 2; range 1 to 4)
Number of DMARDs	1 (1 to 2; range 0 to 5)

Data presented as mean ± standard deviation or *n* (%). DMARD, disease-modifying antirheumatic drug; PASI, Psoriasis Area Severity Index; PsA, psoriatic arthritis; TNFi, tumour necrosis factor inhibitor. ^a^Variables used to construct the propensity score.

These hypotheses include the classical autoimmune basis of disease and the concept that PsA starts at the enthesis with microtrauma which then initiates innate immune events. While not being entirely mutually exclusive, these hypotheses present very different ideas about the initiation/causation of PsA.

### Psoriatic arthritis is an autoimmune disease

Several features in PsA suggest that an autoimmune process may drive the disease. Firstly, susceptibility to develop PsA is associated with class I major histocompatibility complex (MHC) genes [5]. Secondly, CD8^+^ T cells, which present antigen to molecules encoded by HLA class 1 alleles, were shown to predominate in PsA synovial fluid with a reversal of the CD4:CD8 ratio found in rheumatoid arthritis (RA) [6]. Thirdly, CD8^+^ T cells in synovial tissue and fluid obtained from PsA patients are clonally expanded with a reduction in nonspecific T-cell infiltration observed following methotrexate therapy [7,8]. Fourthly, the interesting observation in the setting of advanced HIV disease that both cutaneous psoriasis (PsO) and PsA can occur more frequently and more severely in the setting of CD4^+^ T-cell depletion suggests that persisting memory-effector CD8^+^ T cells (and not CD4^+^ T cells as in RA) drive the disease. Fifthly, PsA developed for the first time after syngeneic bone marrow transplantation from a PsO donor [9]. Finally, there is the response to therapeutic agents directed at activated T cells (for example, cyclosporine, DAB389IL-2 or alefacept), as well as to effector pathways resulting from T-cell activation.

Given the above, one pathogenic hypothesis in PsA is that molecules encoded by certain HLA class 1 alleles bind self-peptides derived from proteins found in entheseal and synovial sites. CD8^+^ T-cell clones specific for these self-peptide–MHC complexes are inappropriately activated, perhaps through the action of dendritic cells and the activated state perpetuated by the continual supply of self-peptides. This is the classic explanation for an autoimmune disease as a consequence of driver clones, and one would expect to recognise one or a few immunodominant T-cell receptor sequences in the inflammatory infiltrate. Clonally expanded T cells have indeed been demonstrated in PsA synovial fluid and tissue; however, despite extensive earlier analysis of the T-cell clonotypes, no evidence for an antigen driving this clonal expansion common to all cases has been identified [8].

Other components of the immune system have an active effector role in disease pathogenesis. Expression of MHC class I chain-related A (MICA) and interleukin (IL)-15 can lead to autoimmunity mediated by CD8^+^ effector T cells, probably due to the capability of IL-15 to drive the expression of the natural killer group 2 member NKG2D [10]. A more recent study demonstrated that PsA patients had upregulated IL-15 and MHC class I chain-related A (MICA) in their affected synovial tissues [11]. This unique inflammatory environment enabled natural killer cell activation and killing via NKG2D and cytosolic phospholipase cPLA2, suggesting a destructive role for natural killer cells when activated by environmental stress signals during the initiation of PsA. Furthermore, as IL-15 could reproduce the phenotype of joint natural killer cells from blood natural killer cells, this study demonstrated that IL-15 is capable of priming resting natural killer cells in tissues to the effector phase. One additional possibility is that the clonally driven CD8^+^ population contains an enriched population of IL-17^+^CD8^+^ T cells in PsA synovial fluid that is not found in RA synovial fluid, suggesting this population of T cells may be worthy of further study [12].

### Psoriatic arthritis is an entheseal-based disease

Ever since the hypothesis published in 1998 [13] and through some elegant supporting anatomical and imaging studies [14], there has been growing support for the concept that the origin of inflammation in spondyloarthritis including PsA is at the enthesis. Enthesitis may be a prominent clinical feature at presentation in up to 38% of PsA patients [15]. Anatomical studies have shown that the enthesis is continuous with the joint structure [14] and that it also merges into the nail bed [16], allowing for the development of concepts that link entheseal involvement with prominent features of PsA, both synovitis and nail dystrophic change. Furthermore, magnetic resonance imaging (MRI) studies have highlighted bone marrow oedema at sites of entheseal attachment in PsA, leading to the hypothesis that it may be microtrauma at entheseal sites that in genetically susceptible individuals leads to inflammation, as evidenced by bone marrow oedema, which then spreads to secondarily involve structures such as the synovium or the nail. Immunohistologic studies that have demonstrated both CD68^+^ macrophages [17] and CD8^+^ T-cell infiltration [18] at sites of entheseal inflammation add further weight to these concepts.

## Review: genetics of psoriatic arthritis

One of the striking features of PsA is that as many as 50% of PsA patients have a family history that includes one or sometimes multiple cases of PsA, PsO, or seronegative spondyloarthritis in their blood relatives, a likelihood 40-fold greater than that of their unaffected spouses [19-21]. Indeed, strong heritability has been demonstrated across four generations in an elegant study [22]. This, along with high twin concordance rates, establishes that germline genes importantly contribute to the definition of the predisposition to develop PsA. While the evidence that genetics plays a major role in who develops PsA has been present for many years, the identification of the genes responsible for conferring PsA has been a lengthy preoccupation for a number of investigators that was limited by the available technology, prevailing diagnostic criteria for PsA and the study size.

### Background findings

Because the highly polymorphic MHC genes have an important role in regulating immune responsiveness and because PsA was considered an immune-mediated disease, MHC genes were the first candidates to be examined for a role in determining susceptibility to the development of PsA, and accordingly this review focuses on the contribution of HLA alleles. Association studies of PsA with MHC genes began with Brewerton’s identification of a significantly increased frequency of HLA-B*27 in individuals with PsA using serologic methods, where it was reported as strongly associated with axial disease [23]. Subsequently, the dominant HLA allele associated with susceptibility to PsO was identified as HLA-C*06:02, as it is now designated, although then it was termed *HLA-Cw6* and found to be present in approximately 60% of PsO cases. As anticipated from the presence of PsO in PsA, PsA was also associated with an elevated frequency of HLA-C*06:02 as first reported in a study using serologic techniques by Murray and colleagues, where C*06:02 was identified in 34.6% of PsA patients, 50% of PsO patients and 13.5% of controls [24]. In later studies, however, the proportion of serologically defined HLA-C*06:02 varied from 60% to levels not significantly different from the frequency in healthy controls (15 to 20%). (For a more detailed summary of the divergent results among these earlier reports, see [25].) The earlier studies were notably limited by the imprecision of the earlier serologic method of determining HLA alleles, by comparing populations that varied in the frequencies of certain HLA alleles and, perhaps most importantly, by the classification criteria for PsA being incompletely delineated. The main alleles and haplotypes associated with PsO and PsA are summarised in Box 1.

### New data concerning psoriatic arthritis genetics

Because of the importance of more precisely determining the role played by HLA alleles in PsA, using improved PsA case ascertainment based on the Classification Criteria for Psoriatic Arthritis (CASPAR) [1] and more accurate methods of allele determination, we set out to compare the similarities and differences in the frequency of HLA-B and HLA-C locus alleles between PsO and PsA. We were particularly interested in addressing the question of the genetic relationship between PsO and PsA and asking whether PsA is a genetically heterogeneous entity [4]. To achieve this, a PsO cohort of 214 patients was assembled from individuals presenting to a dermatologic clinic who were determined to be without features of arthritis, spondylitis or enthesitis through examination by a rheumatologist. This cohort was compared with a PsA cohort (*n* = 359) presenting to a rheumatology clinic, in effect comparing the dermatologist’s perspective of the PsO phenotype with that of the rheumatologist, and asking whether the two perspectives were equivalent. All patients in both cohorts belonged to the relatively homogeneous Irish population and had Irish parents, which diminished the problem of ethnic differences between the cohorts.

**Figure 1:**
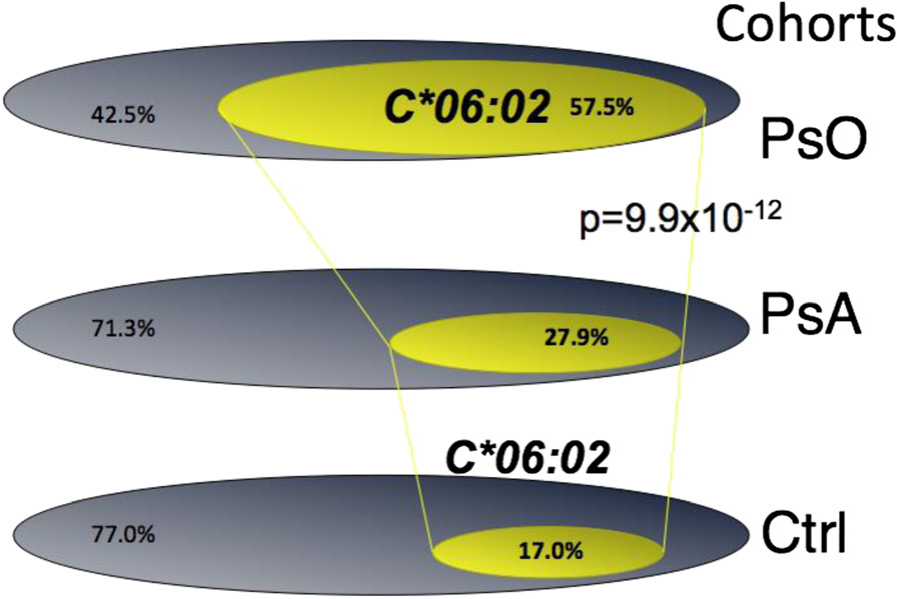
Distribution of HLA-C*06:02:01 among psoriatic arthritis, psoriasis and healthy controls. Venn diagrams in each population illustrate the approximate proportion of individuals that bear the allele HLA-C*06:02:01. Ctrl, control; PsA, psoriatic arthritis; PsO, cutaneous psoriasis.

The first point that emerged from the data analysis addressed the question of whether the distribution of the HLA-C*06:02 alleles in the PsA cohort parallel that found in the PsO cohort (Table 2). As a highly significant difference in the frequency of HLA-C*06:02 was found between PsA and PsO (57.5% vs. 28.7%), we rejected the hypothesis of genetic homogeneity of the PsO phenotype (*P* = 9.94 × 10^−12^), although HLA-C*06:02 was moderately increased in frequency in PsA. Figure 1 shows the differences in distribution of HLA-C*06:02:01 among the three cohorts of PsA, PsO and healthy controls.

**Table 2:** **Distribution of the** HLA-C*06:02 **and** HLA-B*57:01 **alleles in psoriatic arthritis does not parallel that found in cutaneous psoriasis**

**HLA allele**	**PsA (%),** ***n*** **= 359**	**PsA versus control**	**PsO (%),** ***n*** **= 214**	**PsO versus control**	**Control (%),** ***n*** **= 1,000**	**PsA versus PsO**
B*57:01	18.4	2.5 (1.6 to 3.8)	31.3	5.71 (3.9 to 8.3)	7.4	0.30 (0.17 to 0.53)
C*06:02	28.7	1.8 (1.3 to 2.5)	57.5	6.61 (4.18 to 9.1)	19.3	0.26 (0.16 to 0.42)

Data presented as percentage or odds ratio (95% confidence interval). HLA, human leukocyte antigen; PsA, psoriatic arthritis; PsO, cutaneous psoriasis.

Since the frequency of HLA-C*06:02 was appreciably decreased in the PsA cohort compared with that in the PsO cohort, we then asked whether there are other HLA-B alleles that are significantly increased in the Irish PsA cohort. The data showed that HLA-B*27:05:02 and HLA-B*39:01:01 were susceptibility alleles for PsA, but are not high-risk alleles for PsO in the absence of arthritis, and HLA-B*08:01 was associated with PsA susceptibility but appears protective for PsO, being significantly reduced in frequency in PsO (Table 3). This observation emphasised the role of *HLA-B* alleles in PsA susceptibility, a finding supported by the report from Okada and colleagues [26]. Taken together these findings demonstrate that PsA itself is much more genetically heterogeneous than PsO and provide further strong evidence for genetic heterogeneity of the general PsO phenotype. In parallel with our analysis, another group studied this question with similar methods and obtained almost identical results [3,4].

**Table 3:** **Additional HLA-B alleles that are significantly increased or decreased in the psoriatic arthritis cohort**

**HLA allele**	**PsA (%),** ***n*** **= 359**	**PsA versus control**	**PsO (%),** ***n*** **= 214**	**PsO versus control**	**Control (%),** ***n*** **= 1,000**	**PsA versus PsO**
B*27:05	15.6	2.6 (1.7 to 4.2)	4.7	*P* = 0.23	5.5	3.77 (1.9 to 7.6)
B*39:01	6.4	3.5 (1.6 to 7.6)	1.4	*P* = 1	1.2	2.86 (1.1 to 7.6)
B*08:01	37.3	1.6 (1.2 to 2.0)	24.8	0.43 (0.25 to 0.76)	30.1	1.81 (1.6 to 6.5)
B*44:02	14.8	0.53 (0.36 to 0.80)	22.4	*P* = 0.7	24.6	0.60 (0.29 to 0.87)

Data presented as percentage or odds ratio (95% confidence interval). HLA, human leukocyte antigen; PsA, psoriatic arthritis; PsO, cutaneous psoriasis.

**Figure 2:**
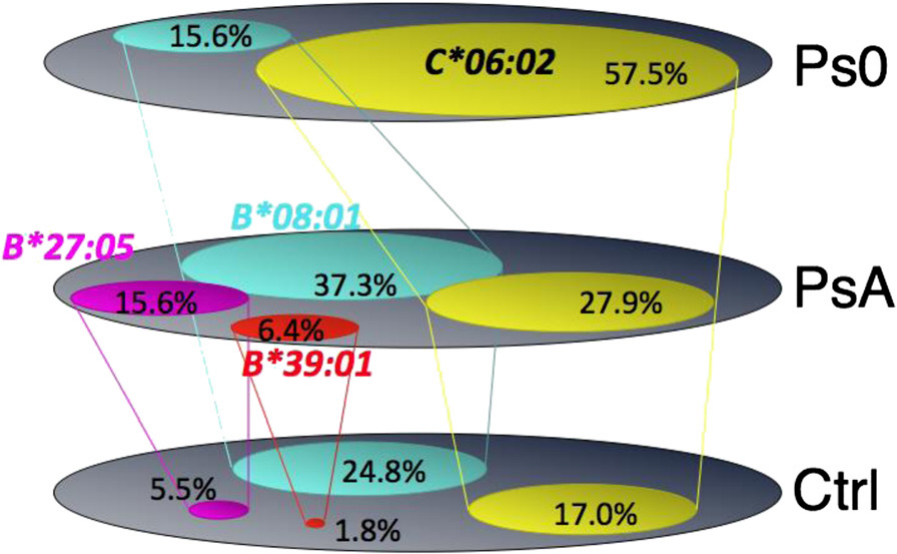
Certain HLA-B alleles in the psoriatic arthritis cohort are significantly increased in frequency compared with their frequency in cutaneous psoriasis and healthy controls. Venn diagrams in each population illustrate the approximate proportion of individuals that bear the alleles. Ctrl, control; PsA, psoriatic arthritis; PsO, psoriasis where there is no arthritis.

The number of different HLA alleles associated with PsA susceptibility, and their differing frequencies between PsA cases and those with isolated cutaneous-only PsO, are illustrated in Figure 2. These studies resolved several important points of divergence in the literature [25]. The genes associated with increased PsA susceptibility (B*08:01, B*27:05, C*06:02, B*39:01 and B*38:01 (not illustrated in the figure)) were almost exclusively present in seven classic ancestral haplotypes (for example, B*08:01-C*07:01, B*27:05-C*02:02, B*27:05-C*01:02, B*37:01-C*06:02, B*57:01-C*06:02, B*39:01-C*12:03 and B*38:01-C*12:03)*.* Apart from C*06:02, which is found on two ancestral haplotypes (B*37:01-C*06:02 and B*57:01-C*06:02), none of the other HLA-B alleles were associated with C*06:02. Additionally, B*44:02-C*05:01 and B*44:03-C*16:01 were associated with significantly decreased susceptibility (not illustrated in the figure), which indicates a protective effect. Table 4 presents the contrasting molecular architecture and peptide binding properties between two HLA-B molecules associated with increased susceptibility and two that appear protective, being associated with significantly decreased susceptibility [4]. The two molecules encoded by B*27:05:02 and B*39:01:01 alleles have negatively charged glutamic acid in the B pocket that preferentially binds the second amino acid side chain (P2) of a peptide, as well as B*08:01 and B*38:01, while the two molecules associated with decreased susceptibility have exactly the opposite charge properties. This interpretation was supported by the recent study from Okada and colleagues in a large cohort of PsA patients using imputed HLA typing of *HLA-B* alleles [26]. This suggests that the particular peptides bound by HLA molecules play an important role in influencing the development of the immune response underlying PsA. Intriguingly, the B*39:06 allele, which differs by only a few amino acids from B*39:01, was not associated with PsA susceptibility, indicating a relatively precise molecular basis for susceptibility.

**Table 4:** **Amino acids in polymorphic sites of HLA-B molecules associated with increased or decreased psoriatic arthritis susceptibility and their contrasting effect on binding peptide P2 side chain anchor amino acids**

**HLA-B molecule**	**P2 pocket amino acid position**		**P2 amino acid binding preference**	**Allele risk estimate** ^**a**^
	**45**	**67**		
B*27:05:02	Glu	Cys	Arg	3.18 (2.14 to 4.71)
B*39:01:01	Glu	Cys	Arg	3.74 (1.99 to 7.01)
B*44:02:01	Lys	Ser	Glu	0.53 (0.38 to 0.74)
B*44:03:01	Lys	Ser	Glu	0.47 (0.24 to 0.92)

Arg, arginine; Cys, cysteine; Glu, glutamic acid; Lys, lysine; Ser, serine. ^a^Data presented as odds ratio (95% confidence interval).

### Different susceptibility genes imply different disease mechanisms, and possibly a different clinical course and therapeutic responses

While the genetic results have implications for who would develop PsA, they formed the basis for asking whether the different genotypes conferred different disease phenotypes. A precedent for an effect of susceptibility alleles on phenotype has been present since the initial study by Brewerton and colleagues where HLA-B*27 was proposed as being linked to the presence of axial disease in PsA [23]. However, this association has been quite inconsistent in subsequent studies [27-31]. Analogously, HLA-C*06:02 was reported to be associated with fewer involved or damaged joints [32].

#### Genotype influences the duration between cutaneous and musculoskeletal involvement

The first clue in the present line of study that different clinical phenotypes may be determined by different genotypes was that the interval between the onset of PsO and the development of PsA was influenced by the patient’s HLA type. The standard teaching is that PsA follows the onset of PsO by 10 to 15 years. We observed that arthritis develops much closer to the appearance of PsO in the HLA-B*27:05:02 or B*39:01:01 subset than in the HLA-C*06:02 subset of PsA cases [4]. This indicated that susceptibility genes were involved in determining the time interval between PsO and PsA and that this time difference is a genetically determined quantitative trait. This suggested that the mechanism of genetic susceptibility in B*27:05:02 individuals resulted in similar penetrance for musculoskeletal and skin disease, and that they were roughly contemporaneous in onset. Indeed nearly one-third of B*27:05:02 patients had the onset of skin disease after the onset of musculoskeletal disease (that is, PsA *sine* PsO). Conversely for HLA-C*06:02 patients, the interval between skin disease and musculoskeletal disease was the expected lag of over a decade and HLA-C*06:02 appeared to specify an effect that included highly penetrant severe cutaneous disease and delayed milder musculoskeletal disease with greatly reduced penetrance.

#### Genetic influence on whether sacroiliitis is symmetrical or asymmetrical

In a follow up to the original genetic paper, 282 patients of the above described genetic study [4] who were available were extensively clinically and radiographically phenotyped, with the assessors completely blinded to the previously reported HLA typing results. We first explored univariate associations of HLA-B and HLA-C alleles and haplotypes with at least 10 occurrences in the cohort, previously associated with susceptibility with individual clinical characteristics using the asymmetric two-tailed Pearson chi-square method [2].

While ankylosing spondylitis is characterised by symmetrical sacroiliitis, in PsA sacroiliitis is more likely to be asymmetric [33]. Table 5 presents data on whether the finding of symmetric or asymmetric sacroiliitis by X-ray imaging is associated with an HLA genotype. The table shows that HLA-B*27:05:02 and both of its haplotypes were strongly associated with symmetrical sacroiliitis. Symmetrical sacroiliitis and B*27:05 was male preponderant (odds ratio = 11.91), resembling the findings in ankylosing spondylitis and supporting current endeavours to group the axial spondyloarthritis together. Both B*27 haplotypes (B*27:05-C*01:02 and B*27:05:02-C*02:02:01) were equivalently associated with symmetric sacroiliitis (Table 6).

**Table 5:** **Frequency of human leukocyte antigen allele or haplotype in forms of sacroiliitis**

**Marker**	**Sacroiliitis**	**Symmetrical sacroiliitis**	**Asymmetrical sacroiliitis**
B*08:01	50.70%	16.70%	62.70%
*P* value	0.006	0.066	<0.000
OR (95% CI)	2.15 (1.06 to 1.77)	0.323 (0.91 to 1.143)	3.8 (1.9 to 7)
B*27:05	23.20%	61.10%	9.80%
*P* value	0.059	<0.000	0.185
OR (95% CI)	1.92 (0.97 to 3.79)	10.63 (3.86 to 29.3)	0.52 (0.19 to 1.39)
C*01:02	18.6%	36.80%	11.80%
*P* value	0.027	<0.000	0.9
OR (95% CI)	2.32 (1.08 to 5.00)	5.57 (2.02 to 15.5)	1.06 (0.41 to 2.76)
C*02:02	10%	26%	4%
*P* value	0.42	0.002	0.26
OR (95% CI)	1.47 (0.57 to 3.76)	5.19 (1.67 to 16.1)	0.43 (0.09 to 1.9)
C*07:01	48.60%	15.8%	60.80%
*P* value	0.119	0.022	0.001
OR (95% CI)	1.53 (0.89 to 2.62)	0.25 (0.07 to 0.89)	1.73 (1.47 to 5.1)
B*08-C*07	47%	16%	59%
*P* value	0.026	0.057	<0.000
OR (95% CI)	1.86 (1.07 to 3.23)	0.31 (0.089 to 1.1)	3.18 (1.7 to 5.92)
B*27-C*01	14.30%	31.60%	7.80%
*P* value	0.064	0.001	0.78
OR (95% CI)	2.2 (0.94 to 5.15)	5.9 (2.0 to 17)	0.85 (0.28 to 2.61)
B*27-C*02	8.6%	26.3%	2.00%
*P* value	0.474	<0.000	0.134
OR (95% CI)	1.44 (0.53 to 3.95)	6.4 (2.0 to 20)	2.4 (0.03 to 1.8)

CI, confidence interval; OR, odds ratio.

**Table 6:** **Enthesitis is preferentially associated with the** B*27:05-C*01:02 **haplotype**

**Marker**	**Enthesitis**
B*27:05	28.10%
*P* value	<0.000
OR (95% CI)	3.65 (1.89 to 7.06)
C*01:02	21.60%
*P* value	<0.000
OR (95% CI)	4.39 (2.02 to 9.57)
C*02:02	10%
*P* value	0.25
OR (95% CI)	1.67 (0.69 to 4.01)
B*27-C*01	17.50%
*P* value	<0.000
OR (95% CI)	4.73 (1.96 to 11.41)
B*27-C*02	9.30%
*P* value	0.213
OR (95% CI)	1.80 (0.71 to 4.59)

CI, confidence interval; OR, odds ratio.

In contrast, asymmetric sacroiliitis, the more prevalent form of sacroiliitis in PsA, was not significantly associated with B*27:05 but, rather, exhibited a strong association with the more prevalent B*08:01-C*07:01 haplotype and its constituent alleles. As a rough estimate of the effect of these two haplotypes on the sacroiliitis phenotype, 24.4% of those bearing B*27:05:02 developed symmetric sacroiliitis and 11.1% asymmetric sacroiliitis, while 2.9% of those with B*08:01 developed symmetric sacroiliitis and 30.8% asymmetric sacroiliitis.

#### Human leukocyte antigen associations of enthesitis

Enthesitis is often a striking feature of PsA. McGonagle and colleagues, based on the finding of prominent bone marrow oedema at sites of ligamentous attachment on MRI scanning, proposed that PsA should be considered an entheseal-based disease with secondary involvement of synovial structures [13]. Subsequent studies confirmed the finding of marrow oedema changes but showed that it is non-PsA specific and is found in only a proportion of PsA patients [34]. We sought to investigate this aspect of PsA in greater depth using a genetic approach, and found that in univariate analysis the haplotype B*27:05-C*01:02 and its two constituent alleles, B*27:05 and C*01:02, were strongly associated with the development of clinically detectable enthesitis in PsA (Table 6). However, neither the associations with the B*27:05:02-C*02:02:01 haplotype or its constituent alleles were similarly significant for the presence of enthesitis. This association suggests that a gene other than HLA-B*27:05, such as C*01:02:01 that is present on the B*27:05-C*01:02 haplotype, drives the association with enthesitis or that a combination of genes on that haplotype confers susceptibility to enthesitis. Furthermore, enthesitis was not significantly associated with the other major alleles implicated in PsA susceptibility. B*08:01 was not associated with enthesitis while enthesitis was inversely associated with C*06:02 (odds ratio = 0.45, *P* = 0.045), indicating that this susceptibility allele is protective for the presence of enthesitis. Accordingly, we conclude that the genetic factors contributing to enthesitis appear distinct from those operating in the development of other features of PsA.

#### Synovial-based phenotypes are associated with B*08:01

In contrast, we found in univariate analysis that the B*08:01:01-C*07:01:01 haplotype and its alleles are not associated with enthesitis, but conversely were associated with more synovial-based pathology such as joint deformity and joint fusion. Joint deformity occurred in 46% of those with B*08, and 86% of those with joint deformity had B*08 (odds ratio = 2.3, *P* = 0.002). Joint fusion was found in 48.8% of those with B*08, and 39% of those with joint deformity had B*08 (odds ratio = 2.0, *P* = 0.008). There was no significant association of joint deformity or joint fusion with B*27:05:02 or C*06:02:01.

#### Dactylitis exhibits a dual association with B*27:05 and B*08:01

Intriguingly, dactylitis was strongly associated with the presence of B*27:05 and particularly the B*27:05-C*01:02 haplotype (Table 7), but not the B*27:05-C*02:02 haplotype, mirroring the haplotype association of enthesitis. However, alone among the phenotypic traits of severe PsA, dactylitis was also positively associated with the B*08:01-C*07:01 haplotype, implying the possibility that a synovial-based process could also result in the phenotype of dactylitis, suggesting heterogeneity in the mechanism underlying this trait. This is fully compatible with the complexity of the process occurring in dactylitis [35-37], including varying contributions of flexor tenosynovitis, synovitis, enthesitis and more circumferential soft tissue oedema. It would be of interest to determine whether stratification of patients by either of these susceptibility alleles resulted in subgrouping patients with different types of digital processes (for example, tenosynovitis/synovitis vs. enthesitis).

**Table 7:** **Dactylitis is associated with both** HLA-B*27:05 **and** B*08:01, **but not** HLA-C*06:02

**HLA allele or haplotype**	**Frequency (%) of HLA**		**Odds ratio (95% CI)**	***P*** **value (two-sided)**
	**With trait**	**Without trait**		
B*27:05:02	21.3	9.8	2.5 (1.2 to 5.0)	0.009
B*27:05:02-C*01:02:01	12.0	5.3	2.5 (1.0 to 6.1)	0.05
B*27:05:02-C*02:02:02	8.7	4.5	2.0 (0.7 to 5.4)	0.2
B*08:01:01	42.7	30.3	1.7 (1.1 to 2.8)	0.03
B*08:01:01-C*07:01:01	42.0	29.3	1.8 (1.1 to 2.9)	0.03
C*06:02:01	25.3	33.8	0.7 (0.4 to 1.1)	0.1
B*44:02:01	9.3	22.0	0.37 (0.2 to 0.7)	0.003
B*44:02:01-C*05:01:01	8.7	18.0	0.43 (0.2 to 0.89)	0.02
B*44:03:01	6.0	15.2	0.36 (0.2 to 0.8)	0.012
B*44:03:01-C*16:01:01	3.3	9.8	0.3 (0.1 to 0.9)	0.03

CI, confidence interval; HLA, human leukocyte antigen.

#### Propensity to develop severe psoriatic arthritis

As an alternative approach to studying genotype–phenotype associations, we constructed a continuous variable severity score to order the cohort based on the presence of one or more of eight distinctive PsA phenotypic characteristics that we considered would denote a more severely involved patient, including enthesitis, symmetric or asymmetric sacroiliitis, dactylitis, joint deformity, joint erosion, joint fusion or osteolysis [2]. This score was used to group patients into three tertiles according to their rank in the severity score. As anticipated, the characteristics that formed the basis of the score, with the single exception of enthesitis, were significantly more prevalent in the group with a severity propensity score in the third tertile compared with those in the first tertile. We then determined the associations of HLA-B and HLA-C alleles and haplotypes with being in the top tertile of the PsA Severity Propensity Score, developing an HLA risk score in which +1 was awarded to the haplotypes B*27:05-C*02:02, B*37:01-C:06:02 and B*08:01-C*07:01 while haplotypes including B*44:02 or C*07:02:01 were awarded –1 and HLA_B*57:01-C*06:02 was awarded 0. Since the genotype of an individual contains two haplotypes, the risk score ranged from –2 to +2, allowing evaluation of the contribution of the alleles on both maternal and paternal chromosomes to disease phenotype. Each unit increase in this adjusted score, on average, was associated with a 168% higher odds of being in the most severe PsA tertile (odds ratio = 2.68, *P* <0.001). The additivity of the scores indicated that B*27:05-C*02:02, B*37:01-C*06:02 and B*08:01-C*07:01 were each equivalently associated together with a more severe phenotype, and this was counterpoised by the haplotypes associated with a zero or negative weight. Intriguingly, these findings appear to suggest that the presence of B*37 on a haplotype containing C*06:02 modifies the action of C*06:02, a point also raised by Okada and colleagues [26].

However, the haplotype B*27:05-C*01:02, which was associated with enthesitis in univariate analysis, was not associated with an elevated severity propensity score. This indicates an unlinking of enthesitis with other elements that were studied in the PsA phenotype. Taken together, these findings suggest that there are distinct genetic factors contributing to enthesitis and these are different from those operating in the development of most other features associated with PsA.

## Conclusions

### Implications of newer findings on concepts of genotype–phenotype relationships

There are several implications for the finding that certain HLA alleles and, most strikingly, particular haplotypes that are implicated in the susceptibility to develop PsA contribute differentially and often additively to the magnitude of different traits comprising the phenotype of PsA [2]. Despite the relatively large size of this cohort, with 282 PsA cases clinically phenotyped and sequence-based HLA typed to at least four digits, the study must be considered exploratory and requires validation in different ethnic groups, especially those with a greater admixture of populations having different principal ancestral haplotypes. In this way, the association of a particular allele within the context of a given haplotype can be better determined. One anticipates that there will be different genotype–phenotype associations in a population that does not include the major Northern European haplotypes such as B*08:01-C*07:01, or those involving B*27. The disparate phenotypic associations of different alleles/haplotypes emphasise the distinct contribution of each different susceptibility allele; however, the overarching common features of all PsA patients may depend on the common molecular architecture of susceptibility molecules (Table 5), supported by the recent report of Okada and colleagues [26]. Apart from the general mechanistic interpretation of these genotype–phenotype associations that different HLA alleles bind different self-peptides and drive differing autoimmune responses directed to distinct target molecules, at this stage of knowledge it is not possible to speculate on the precise molecular mechanisms that may be operating to result in a different clinical phenotype. It is possible that some inflammatory pathways will be more analogous to those in PsO, while others may resemble those of ankylosing spondylitis.

It is interesting to note that the newer genetic findings are consistent with and appear to provide a molecular explanation for the long-held clinical observations of Moll and Wright [38], identifying entheseal predominant, synovial predominant and axial predominant as three of the major forms of PsA, with each now found to have differing HLA associations. The findings summarised in this review are also relevant to the discussion of pathogenic mechanisms in PsA.

First, since different HLA molecules bind different peptides, they probably imply the presence of distinct autoimmune responses that are driving each of the phenotypes separately associated with different HLA alleles. As a corollary, the different peptides suggest that different proteins, perhaps with a different cellular distribution, are targets of these responses. Indeed, the finding that several different HLA alleles confer susceptibility to PsA is an argument in favour of an autoimmune hypothesis, since it directs attention to the different peptide binding properties of the molecules encoded by the susceptibility alleles.

Secondly, one anticipates that these responses may be further distinguished by different predominant mechanisms that use one or another cytokine, and so forth, perhaps indicating that different drugs and biologics may be effective in different genetic subsets. These data also raise the possibility that there is a predominant type of target tissue involved in each separate process. For example, it may prove to be the case that whether you have HLA-B*27:05 or HLA-B*08:01 will determine whether you have symmetrical or asymmetrical sacroiliitis or whether you have a disease that is more axial or entheseal predominant, or perhaps more synovial predominant. The question of how these genotypes might result in a particular clinical phenotype is unclear, but the work of Sherlock and colleagues would suggest that in a mouse model the development of enthesitis may be linked to the presence of a particular subset of T cells (IL-23R^+^, CD3^+^CD4^−^CD8^−^). IL-23 elaborated at the enthesis through an effect of HLA-B*27 on a haplotype encoding C*01:02 acts on this entheseal cell population to elaborate inflammatory mediators including IL-6, IL-17 and IL-22 [39]. Furthermore, their data suggest that the presence of these entheseal resident cells and their production of IL-22, which activates signal transducer and activator of transcription 3-dependent osteoblast-mediated bone remodelling, explains why dysregulation of IL-23 results in inflammation at this precise anatomical site. This dysregulation of IL-23 is possibly determined at a genetic level as a result of the response of HLA-B*27 to microtrauma. Whether a similar model applies to human disease, or even a proportion thereof where there is prominent entheseal involvement (<30% of PsA cases), has yet to be demonstrated.

**Figure 3:**
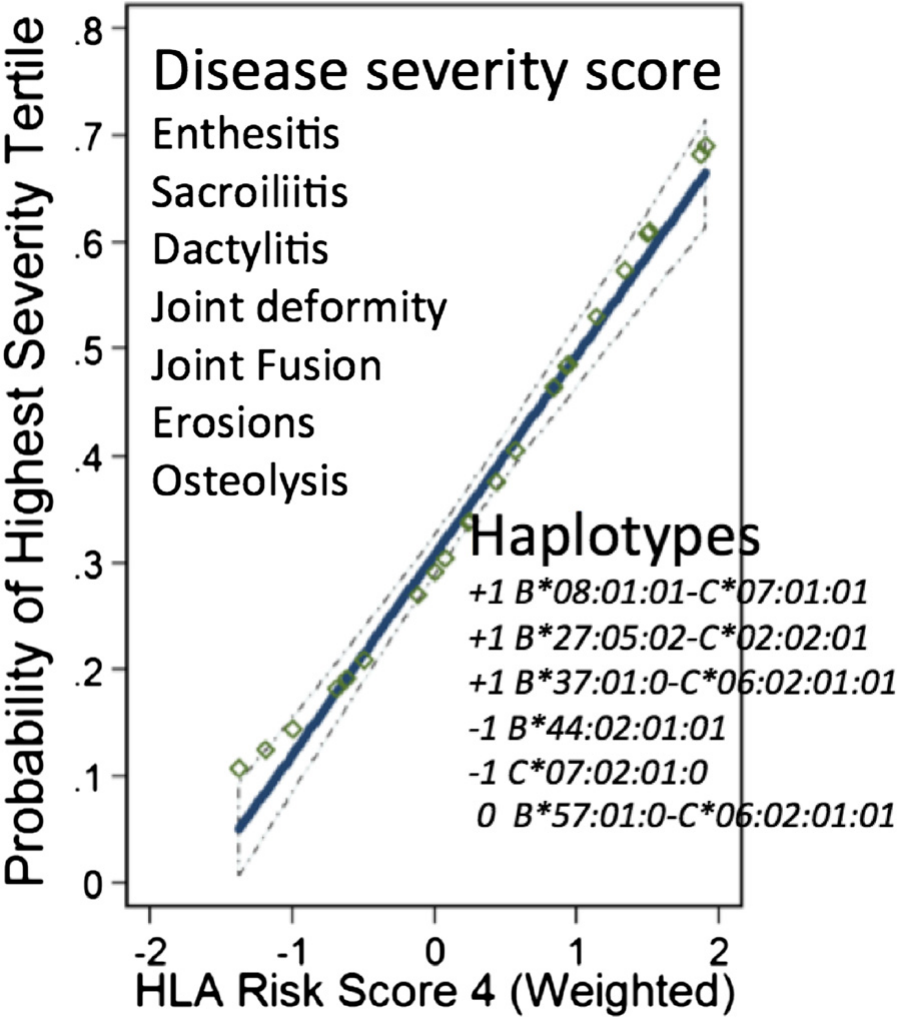
Contribution of positive and negative risk alleles on both chromosomes to psoriatic arthritis severity. An additive model including all genotypes positively or negatively associated with the propensity to develop severe psoriatic arthritis resulting in a composite human leukocyte antigen (HLA) risk score.

However, the present findings argue against the enthesitis hypothesis as a fundamental explanation for PsA in all cases. The data summarised in Table 7, showing that enthesitis is preferentially associated with the B*27:05-C*01:02 haplotype, are not consistent with enthesitis being common to all PsA cases. Furthermore, in the development of the propensity score (Figure 3) the presence of enthesitis was not significantly associated with the other traits comprising the score measuring the propensity for severe PsA [2]. Furthermore, prominent entheseal involvement on MRI is seen in only a minority of patients. Indeed, one study found that MRI was not able to differentiate at the group level between early PsA and RA on the basis of entheseal-related disease [34]; however, a subgroup of PsA patients had diffuse extracapsular enhancement (30%) or diffuse bone oedema (20%). In addition and of interest [40], HLA-B27 positivity in PsA and in axial spondyloarthritis defined a group of patients with more severe axial bone marrow oedema on MRI that is probably related to the classic ankylosing spondylitis phenotype, while HLA-B27-negative PsA was likely to be reported as a negative MRI examination result.

For the two-thirds of PsA patients that appear to have a more synovial-predominant pattern of disease and who are likely to be HLA-B*08:01, a more conventional autoimmune basis of disease may apply. It is certainly possible in these subjects that molecules encoded by HLA class 1 alleles are recognising self-peptides derived from proteins found in synovial sites. CD8^+^ T-cell clones specific for these self-peptides would be inappropriately activated, perhaps by dendritic cells, and the activated state perpetuated by the continual supply of self-peptides. These interactions may result in the elaboration of cytokines such as interferon gamma or tumour necrosis factor alpha, which in turn drive the inflammatory cascade.

Such an explanation of disease pathogenesis goes some way to explain the consistent occurrence of dichotomous therapeutic responses whereby skin and articular structures, for example, respond with different magnitude or even direction to a given therapeutic agent (for example, cyclosporine, ustekinumab, IL-17 inhibition). Using the primary outcome measure of the American College of Rheumatology 20% (ACR20) improvement in clinical response, there is also a consistent degree of nonresponse to any therapeutic agent (30 to 50%) – suggesting that patients with certain clinical features will respond whereas other patients with other features will not. These observations are consistent with the hypothesis that discreet inflammatory pathways may operate across different tissue compartments.

Finally, we would propose a testable hypothesis whereby PsA can be considered to have at least four clinical phenotypes – synovitis predominant, entheseal predominant, axial predominant and mutilans – all determined by genotype. In each of these phenotypes and perhaps as a consequence of the inflammatory events that occur following the recognition of specific peptides presented by molecules encoded by susceptibility allotypes and recognised by specific T-cell clonotypes, a different pattern of inflammation involving diverse immunoreactant molecules and mediators such as cytokines may be unleashed that help to determine clinical disease expression. As mentioned, this is a testable hypothesis with RNA genome sequencing and protein and cytokine profiling of very well-characterised clinical phenotypic/genotypic groupings likely to reveal consistent differences between the genotypic groupings.

## Box 1. The most common human leukocyte antigen alleles and haplotypes implicated in psoriatic arthritis susceptibility and their location within the major histocompatibility complex

## AllelesAncestral extended haplotypes

Increased susceptibility

HLA-C*06:02:01B*37:01-C*06:02, B*57:01-C*06:02

HLA-B*27:05:02B*27:05:02-C*01:02, B*27:05:02-C*02:02

HLA-B*08:01B*08:01-C*07:01

HLA-B*38:01B*38:01-C*12:03

HLA-B*39:01B*39:01-C*12:03

Increased susceptibility

HLA-B*44:02:01B*44:02-C*05:02

HLA-B*44:03:02B*44:03-C*16:01

## Abbreviations

HLA: human leukocyte antigen
IL: interleukin
MHC: major histocompatibility complex
MRI: magnetic resonance imaging
PsA: psoriatic arthritis
PsO: cutaneous psoriasis
RA: rheumatoid arthritis
